# Virological profile of pregnant HIV positive women with high levels of CD4 count in low income settings: Can viral load help as eligibility criteria for maternal triple ARV prophylaxis (WHO 2010 option B)?

**DOI:** 10.4314/pamj.v10i0.72239

**Published:** 2011-10-25

**Authors:** Anne Esther Njom Nlend, Chantal Same Ekobo, Suzie Tetang Ndiang Moyo, Gisele Chewa Nguetcheng, Peter Ngang, Sidonie Lyeb, Luc Meka, Martin Baane

**Affiliations:** 1 National Social Insurance Fund Hospital, Yaoundé, Cameroon; 2 Association Camerounaise d'Aide aux Personnes et Familles Affectées par le SIDA (ACAPFAS), Yaoundé, Cameroon

**Keywords:** Pregnant women, HIV positive, viral load, CV4, low income, virology profile, ARV, Cameroon

## Abstract

**Introduction:**

The objective of the study was to determine HIV-1 RNA load profile during pregnancy and assess the eligibility for the maternal triple antiretroviral prophylaxis. It was an observational cohort of pregnant HIV positive women ignorant of antiretroviral therapy with CD4 cell count of>350/mm^3^

**Methods:**

Routine CD4 cell count assessment in HIV positive pregnant women completed by non exclusive measurement of the viral load by PCR /ARN in those with CD4 cell count>350/mm^3^. Exclusion criteria: highly active antiretroviral therapy prior to pregnancy.

**Results:**

Between January and December 2010, CD4 cell count was systematically performed in all pregnant women diagnosed as HIV-infected (n=266) in a referral center of 25 antenatal clinics. 63% (N=170) had CD4 cell count>350/mm^3^, median: 528 (IQR: 421–625). 145 underwent measurement of viral load by PCR/RNA at a median gestational of 23 weeks of pregnancy (IQR: 19–28). Median viral load 4.4log_10_/ml, IQR (3.5–4.9).19/145(13%) had an undetectable viral load of=1.8log_10_/ml. 89/145(61%) had a viral load of=4 log_10_/ml and were eligible for maternal triple ARV prophylaxis.

**Conclusion:**

More than 6 in 10 pregnant HIV positive women with CD4 cell count of>350/mm^3^ may require triple antiretroviral for prophylaxis of MTCT. Regardless of cost, such results are conclusive and may be considered in HIV high burden countries for universal access to triple antiretroviral prophylaxis in order to move towards virtual elimination of HIV MTCT.

## Introduction

Sub-Saharan Africa continues to record the highest burden of the HIV-1 epidemic, contributing more than 90% in the new cases of perinatally acquired HIV pediatric infections [1]. Cameroon unfortunately is listed among the 20 countries most affected by the HIV pediatric pandemic given the number of HIV pregnant women expected yearly [2]. Mother-to-child transmission of HIV is known to be correlated to maternal viral load, and maternal HIV-1 RNA load has been shown to be the strongest predictor of vertical transmission. In fact, the rate of MTCT has been estimated at 0.3% when maternal viral load is<1000 copies/ml), at 3% between 1000–10000 copies /ml and at 7% when it is>10000 copies/ml [3–5]. Despite such evidence, maternal viral load has never been systematically measured in pregnant women in Cameroon, neither to determine the burden of primary acute infection in pregnancy nor to determine the best antiretroviral protocol. The aim of this study was to describe the virological profile of pregnant women newly diagnosed as HIV infected and unaware of treatment.

## Methods

### Study Population and Procedures

The study population was an observational cohort of pregnant HIV positive women. Pregnant women with<36 weeks of amenorrhea were enrolled in 25 antenatal clinics in the Djoungolo Health District. After opt-out counseling, serial HIV-1/2 algorithm antibody tests were done using Determine HIV-1/2 assay(Abbott Diagnostics, Hoofddorp, the Netherland) and SD bioline HIV 1/3 3.0 (Standard diagnostics Inc, Kyonggi-do, South Korea) rapid kits on mothers' serum samples. For all HIV positive women, CD4 cell counts were measured in the referral laboratory. The method used a flow cytometer for measuring the percentage of CD4 T cells; an automated blood cell counter helped to measure the total number of lymphocytes. Results were produced in absolute numbers and in percentages. CD4 cell count screening was followed by non exclusive measurement of viral load when CD4 cell counts were>350/mm^3^. Maternal baseline serum samples were quantified for HIV-1 RNA load using real Time reverse transcriptase PCR (Generic HIV viral load, Biocentrics). All the samples were analyzed with the Laboratoire Centre Pasteur in Yaoundé acting as service provider at a unit cost of 30US$. ZDV was started for all the others non eligible for triple antiretroviral treatment at 14 weeks of pregnancy, while waiting for the result of the viral load.

### Data collection and Statistical Analysis

The following qualitative variables were considered: maternal age, WHO clinical stages. Quantitative variables included: CD4 cell count and HIV Viral load. Viral load values were log10 transformed and stored in an Excel sheet form. A threshold of≤1.8 log_10_ was considered as undetectable viral load. For this descriptive analysis we considered (frequency, percentage, medians with their quartile). A confidence interval of 95% was considered as the margin for percentage accuracy.

### Ethics and administrative aspects

All interventions within this project were implemented under the supervision of the District Office of the Ministry of Public Health. Data collection and monitoring of the intervention was part of the routine activities of the Health District. Free measurement of HIV1 RNA viral load was offered to all the eligible mothers at the referral center where all activities were coordinated by the Center for HAART treatment and the local board in charge of fighting against HIV and AIDS.

## Results

### Demographic characteristics of the pregnant women

Mothers′ mean age (SD) was 27.0 years IQR 22.5–30 and 15/172(8.7%) were adolescents. All the mothers were classified in WHO clinical stage 1 or 2.

### Immunological profile

Median CD4 cell count was 534 cell/mm^3^, IQR (442–646) and 68/170 had CD4 cell counts of between 350 and 500/mm^3^.

### Virological profile

Maternal viral load and transmission

Table 1 resumes the distribution of the viral load of our positive pregnant women. 126/145 (87%) had detectable serum HIV-1 RNA loads ranging from 1.9 to 6.5 log_10_. The median log_10_ viral load was 4.4(IQR 3.5–4.9). 13%, 95% CI (7.5–18) had an undetectable viral load. A viral load of>3log_10_ was recorded for 107/145(74%); 89/145(61%) had a viral load of≥4log_10_ and for 29/145 (20%, 95% CI 13.5–26.5) a very high level of viral load as usually observed during primary acute infection, was noted (≥5log_10_).

**Table 1:** Distribution of HIV-1 RNA load in HIV positive pregnant women with CD4 cell count >350/mm^3^

Viral load in log _10_ copies/ml	Number of pregnant women	% of pregnant women	95% Confidence interval
Undetectable	19	13	7.5–18
1,9–3,9	37	26	19–33
≥4	89	61	52–70
Total	145	100	

## Discussion

To our knowledge, this is the first study in Cameroon where viral load is systematically determined in pregnant women in order to assess the eligibility to more effective antiretroviral regimen especially at high levels of CD4 cell count. Our key finding is that the level of HIV viral load is≥4log 10/ml in more than 60% of HIV pregnant women, ignorant of HAART, with CD4 cell count>350/mm^3^, making them eligible for maternal triple ARV prophylaxis as required elsewhere. Taking into account that an average of 4 in 10 HIV positive pregnant women are yet to be eligible for HAART because of CD4 cell count of<350/mm^3^, it may be deduced that among the remaining 6 in 10, at least 4 may require HAART for prophylaxis according to their HIV viral load. Altogether 8 in 10 women at least may be eligible for HAART during pregnancy based upon biological criteria. Such results vindicate the opinion of those who claim that universal access to maternal triple ARV prophylaxis during pregnancy regardless of CD4 cell count is a must and are consistent with the latest WHO recommendations for women not yet in need of treatment for themselves, namely option B [6–8].

In Sub-Saharan Africa, some countries have opted for triple antiretroviral therapy for all pregnant women according to the level of care [9]. In the dream cohort, generalizing access to such an approach has led to virtual elimination of MTCT [10]. Option B is recommended without HIV RNA viral load as a prerequisite, but unfortunately many countries affected by generalized epidemic have not been able to opt for such an approach due to cost implications. In the context of option A, access to HIV viral load even at cost recovery rate could help for better management of the target population, notably in HIV care and treatment referral centers.

## Conclusion

We concluded that antenatal serum HIV-1 RNA viral load, stands above 4log_10_ in more than 60% newly diagnosed pregnant women in Cameroon with high levels of CD4 cell count. Such results suggest the promotion of the routine measurement of viral load during pregnancy in A option country or the switch to B option for all, in order to ensure larger access to maternal antiretroviral triple prophylaxis. This approach is likely to be more effective in lowering maternal viral load and reducing mother-to-child transmission of HIV.
